# Gaze cuing of attention in snake phobic women: the influence of facial expression

**DOI:** 10.3389/fpsyg.2015.00454

**Published:** 2015-04-20

**Authors:** Carolina Pletti, Mario Dalmaso, Michela Sarlo, Giovanni Galfano

**Affiliations:** ^1^Department of General Psychology, University of Padova, Padova, Italy; ^2^Department of Developmental and Social Psychology, University of Padova, Padova, Italy; ^3^Center for Cognitive Neuroscience, University of Padova, Padova, Italy

**Keywords:** gaze cuing, snake phobia, facial expression, spatial attention, emotion

## Abstract

Only a few studies investigated whether animal phobics exhibit attentional biases in contexts where no phobic stimuli are present. Among these, recent studies provided evidence for a bias toward facial expressions of fear and disgust in animal phobics. Such findings may be due to the fact that these expressions could signal the presence of a phobic object in the surroundings. To test this hypothesis and further investigate attentional biases for emotional faces in animal phobics, we conducted an experiment using a gaze-cuing paradigm in which participants’ attention was driven by the task-irrelevant gaze of a centrally presented face. We employed dynamic negative facial expressions of disgust, fear and anger and found an enhanced gaze-cuing effect in snake phobics as compared to controls, irrespective of facial expression. These results provide evidence of a general hypervigilance in animal phobics in the absence of phobic stimuli, and indicate that research on specific phobias should not be limited to symptom provocation paradigms.

## Introduction

Highly anxious people show a preferential allocation of attentional resources to stimuli representing threats, even in the absence of actual danger, and suffer from general hypervigilance aimed at quickly identifying potential threats (Mathews and MacLeod, 1994; Williams et al., 1997; Bar-Haim et al., 2007; Cisler and Koster, 2010). This attentional bias to threat is thought to play a role in both the etiology and maintenance of the disorder (Bar-Haim et al., 2007; Cisler and Koster, 2010), a hypothesis that has been supported by empirical studies that show how anxiety vulnerability increases after the induction of an attentional bias for threat and decreases after a task designed to reduce this bias (MacLeod et al., 2002; Amir et al., 2008; Hakamata et al., 2010; Heeren et al., 2012a).

Research on attentional biases in anxiety disorders has also focused on animal phobia, a disorder consisting of a persistent and unreasonable fear of a certain animal species (American Psychiatric Association, 2000). Individuals with animal phobia suffer from a specific form of attentional bias, as their attention is preferentially directed toward phobia-related stimuli rather than toward threatening stimuli in general (Mogg and Bradley, 2006; Öhman, 2008). So far, only a few studies have investigated biases for non-phobic (NP) stimuli in animal phobia. Among these, a number of studies reported faster reaction times (RTs) and enhanced early event-related potentials (ERPs) in animal phobics, as compared to control participants, not only to phobic stimuli (i.e., spiders or snakes), but to control stimuli as well (i.e., flowers or birds), a result that has been interpreted as indicating a general hypervigilance in early processing stages in specific phobia (Kolassa et al., 2005, 2006). However, these results could be ascribed to anticipatory anxiety, because participants were expecting fear-related stimuli to appear.

Interestingly, a functional magnetic resonance imaging (fMRI) study (Wright et al., 2003) reported a processing bias for emotional faces in animal phobics in a task in which no phobic stimuli were presented. When presented with fearful faces, phobic individuals, as compared to control participants, exhibited higher activity in brain areas such as the right insula that are known to be involved in generating fear responses to disorder-related stimuli. Still, these results do not allow us to understand whether such bias was specific for the expression of fear, because fear was the only emotional expression presented in this study. To clarify this issue, Sarlo and Munafò (2010) conducted an ERP study and compared processing of fearful, disgusted, neutral and angry facial expressions. The results showed that snake phobics (SP), compared to NP participants, showed a lower P200 amplitude for expressions of fear and disgust, and a relatively greater positivity in the subsequent time window for negative expressions as compared to neutral faces. Given that P200 is an attention-related index of fast automatic detection of emotionally salient stimuli, Sarlo and Munafò (2010) interpreted this pattern as evidence of an initial cognitive avoidance followed by a hypervigilant processing mode. They also argued that such an effect may reflect these expressions’ ability to signal the presence of a phobic threat in the environment. In this regard, it is widely recognized that emotional faces are rapidly processed (Batty and Taylor, 2003) and, together with gaze direction, allow us to understand where someone’s attention is directed, thus providing information on relevant changes in the environment (Emery, 2000; Nummenmaa and Calder, 2009; Bayliss et al., 2010; Graham et al., 2010; Yiend, 2010). As fear and disgust characterize the emotional states experienced by animal phobics during exposure to the phobic object (de Jong et al., 2002; Schienle et al., 2005; Olatunji et al., 2007), a disgusted or fearful expression could imply that the probability that the feared animal is nearby is increased and that the surroundings are no longer safe. This, in turn, could trigger a hypervigilant processing mode (Sarlo and Munafò, 2010).

Provided that a constant monitoring of the environment for threat is a main feature of clinical anxiety (Mathews and MacLeod, 1994; Williams et al., 1997), it could be hypothesized that animal phobics are subject to a similar monitoring, aimed at locating the possible presence of the feared animal in the environment rather than a generic threat. Indeed, specific phobics show hypervigilance and monitoring of the environment when they expect the phobic object to appear (Straube et al., 2007). It is possible that this hypervigilance could also be automatically triggered by salient stimuli signaling the presence and the location of a possible threat, even in contexts where no phobic stimuli are about to appear. The finding of a processing bias in animal phobia in the absence of phobic stimuli could have important implications for research on this disorder and for its treatment, as involuntary orienting of attention and excessive scanning of the environment may contribute to maintainance of the disorder, as is the case for other types of anxiety disorders such as generalized anxiety disorder and social phobia (Mogg and Bradley, 1998, 2005; MacLeod et al., 2002; Heeren et al., 2012a). Here, we aimed to further explore the effects of emotional expressions on the allocation and orienting of attentional resources in animal phobics, as these stimuli are frequently encountered in everyday life.

The impact of emotional expressions on attentional orienting has been widely studied using the gaze-cuing paradigm, in which a naturally salient stimulus, i.e., another person’s gaze, produces a shift in participants’ attention. In this paradigm, a face is presented at the center of the screen with the eyes averted leftward or rightward. After a variable time interval (stimulus onset asynchrony, SOA), a peripheral target randomly appears to the left or to the right of the face. Typically, lower RTs are observed for targets appearing in gazed-at rather than non-gazed-at locations, a result known as gaze-cuing effect (Friesen and Kingstone, 1998; Driver et al., 1999; Dalmaso et al., 2013), which is interpreted as evidence that gaze direction can elicit a corresponding shift of attention in an observer (see Frischen et al., 2007, for a review).

Several studies have hypothesized the existence of an emotional modulation of gaze-cuing effects, but the available evidence is mixed (Hietanen and Leppänen, 2003; Holmes et al., 2010; Galfano et al., 2011; Rigato et al., 2013; but see Pecchinenda et al., 2008; Itier and Batty, 2009; Kuhn and Tipples, 2011; Lassalle and Itier, 2013; Neath et al., 2013). The results seem to be more consistent when trait anxiety or trait fearfulness of the participants is taken into account. Individuals with high anxiety or high trait fearfulness show a stronger cuing effect for fearful faces as compared to neutral and happy faces (Mathews et al., 2003; Tipples, 2006), and individuals with high anxiety also show a smaller cuing effect for angry faces as compared to neutral and happy faces (Fox et al., 2007). Hence, emotional gaze-cuing paradigms are perhaps not sensitive enough to reveal variations in attentional responses to gaze as a function of different emotional expressions in the general population, but seem to be apt at highlighting differences between the general population and people who are more prone to experience fear or anxiety.

On these grounds, we reasoned that the gaze-cuing paradigm could represent a useful tool to investigate how facial expressions influence orienting of attention in animal phobics. We focused on fear and disgust as these emotions are particularly relevant in the context of animal phobia (e.g., de Jong et al., 2002; Olatunji et al., 2007) and hypothesized that these expressions would elicit an enhanced gaze-cuing effect in animal phobics, as they may signal the presence of a phobic threat in the environment (Sarlo and Munafò, 2010). To discern specific effects of phobia-related expressions from effects due to negative valence, we included angry faces as control stimuli. We employed dynamic face cues and a morphing technique aimed at producing a more ecologically valid paradigm (Putman et al., 2006; Tipples, 2006; Uono et al., 2009; Bayless et al., 2011), in which emotional expressions appeared after the change in gaze direction, simulating a realistic emotional change of expression due to a stimulus in the surroundings. To investigate the time course of the emotional influence on the gaze-cuing effect, two different SOAs were used: a 200-ms SOA, in order to tap into exogenous processes, and a 500-ms SOA, in order to investigate later, more controlled stages of processing (Müller and Rabbitt, 1989). We did not include threatening stimuli in the experiments because we aimed to address the presence of attentional bias in animal phobics in a context in which no anticipation of the phobic object occurred.

## Materials and Methods

### Participants

Participants recruited at the University of Padova were selected through the administration of the Snake Questionnaire (SNAQ; Klorman et al., 1974), a self-report questionnaire assessing the severity of snake fear and avoidance. The SP group was composed of individuals scoring ≥18 (corresponding to the 85th percentile calculated on a sample of 496 female students), the NP control group was composed of individuals scoring ≤10 (the 50th percentile). To confirm the presence of snake phobia in the SP group and the absence of specific fears in the NP group, all participants underwent a semi-structured interview based on the DSM-IV criteria for specific phobia (Brown et al., 1994). The Trait version of the State-Trait Anxiety Inventory (STAI-Y2, Spielberger et al., 1970) was also administered, as the literature suggests that anxiety influences the emotional modulation of gaze-cuing of attention (Mathews et al., 2003; Fox et al., 2007). Given the higher prevalence of specific phobias in females (American Psychiatric Association, 2000), only women were selected for the present study. Twenty female SP and 20 female NP participants took part in the experiment. One participant in the SP group presented RTs greater than two standard deviations above the mean in every experimental condition and was excluded from the analyses. The final sample was composed of 19 SP (*M*_age_ = 22 years, SD = 2.03, *M_STAI-Y2_* = 45.37, SD = 9.54, *M_SNAQ_* = 21.10, SD = 3.36, range = 18–28) and 20 NP individuals (*M*_age_ = 22 years, SD = 1.29, *M_STAI-Y2_* = 35.65, SD = 8.17, *M_SNAQ_* = 5.15, SD = 2.89, range = 1–10).

All participants were naïve to the purpose of the experiment and reported normal or corrected-to-normal vision. This research had been approved by the local ethics committee and was performed in accordance with the ethical standards laid down in the 1964 Declaration of Helsinki and its later amendments. All persons gave their informed consent prior to their inclusion in the study.

### Stimuli

From the NimStim set of facial expression (Tottenham et al., 2009), color pictures of six individuals, three males (codes: 22M, 34M, 26M) and three females (codes: 1F, 6F, 9F), portraying four prototypical expressions each (disgust, fear, anger, and neutral) were randomly selected. Only photographs of Caucasian individuals were included, as participants were all Caucasian and we wanted to rule out the potential effect related to using faces belonging to different ethnic groups (Pavan et al., 2011). Each picture was modified to create two additional pictures with eyes averted left or right. Facial expressions of intermediate intensity (55%) were created using MorphMan 2000 software (STOIK Imaging, Moscow, Russia): for each face with averted gaze, an intermediate picture between the neutral and the emotional expression was created. We did not include a condition in which the final emotional expression was neutral, because the absence of any expression changes and face movements would have made it inappropriate as a control condition. The faces were presented on a gray background and measured approximately 17.5 cm × 20.5 cm (19.1° × 24.5°). To increase ecological validity, non-facial features (i.e., neck and hair) were not removed. Target stimuli were “L” and “T” letters, measuring 0.7 cm × 0.7 cm (1.2° × 1.2°), presented 14.5 cm (8.56°) leftward or rightward from the center of the screen and vertically aligned with the eyes. The fixation cross measured 0.7 cm × 0.7 cm (1.2° × 1.2°). Stimuli were presented at a distance of 80 cm on a 16-inch monitor (640 × 480 pixels, 60 Hz) using E-Prime 1.2 (Psychology Software Tools, Pittsburgh, PA, USA).

### Procedure

**FIGURE 1:**
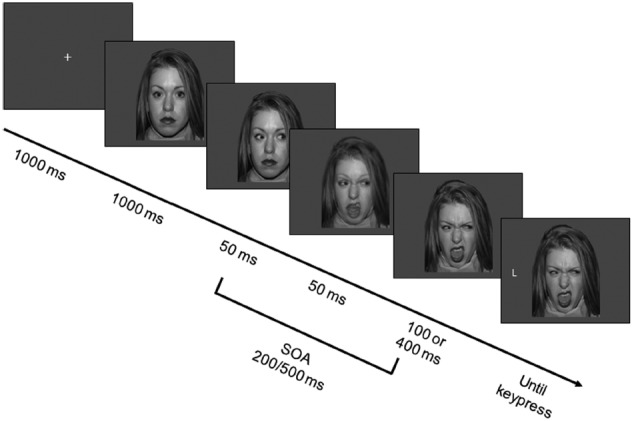
**Sequence of events in the experiment.** Each trial started with a fixation cross, which was replaced after 1000 ms by a neutral face gazing straight ahead. After 1000 ms the eyes moved either rightward or leftward. After 50 ms the facial expression changed to an intermediate one, and after 50 ms to a disgusted, angry or fearful one. After either 100 or 400 ms, a target appeared and participants had to press a different key depending on whether the target letter was a T or an L. In the example, the face portrays a disgusted expression and the target is spatially incongruent to the gaze-cue. Note that the SOAs, that is the time intervals between eye movement and target appearance, are 200-ms and 500-ms. Stimuli are not drawn to scale. Color stimuli were used.

Each trial began with the presentation of a fixation cross at the center of the screen, which lasted 1000 ms and was then replaced by a face bearing a neutral expression with direct gaze. After 1000 ms, the face was replaced by the same picture with eyes averted either leftward or rightward. After 50 ms, the facial expression of intermediate intensity was presented for 50 ms, and then replaced by the full-intensity facial expression. Thus, the facial expression would appear to participants to have changed promptly and dynamically after the eye movement. The target letter appeared leftward or rightward of the face after either 100 or 400 ms, resulting in two different SOAs (200 ms vs. 500 ms from gaze cue to target; see Figure 1). Participants performed a discrimination task by pressing one of two horizontally aligned keys (“D” and “K,” labeled with two differently colored labels). They were instructed to respond as quickly and accurately as possible while maintaining central fixation. Response mapping was counterbalanced across participants. Participants were asked to ignore the gaze direction because it did not predict the target location.

A practice block of 16 trials was followed by two experimental blocks of 144 trials each in which all the conditions were randomly presented for an equal number of times. The whole experiment lasted about 40 min.

## Results

Results are reported with η^2^ effect sizes and their 90% confidence interval, as recommended by Steiger (2004).

Snake phobics participants were significantly more anxious than NP individuals, *t*(37) = –3.42, *p* = 0.001, η^2^ = 0.24, 90% CI [0.06, 0.41]. Because of this finding, the data were submitted to an analysis of covariance (ANCOVA), in order to control for possible effects of anxiety.

Prior to RT analysis, incorrect responses (4.5% of all responses) and RTs greater than 1500 ms or smaller than 150 ms were removed (0.2% of correct responses). Mean RTs for correctly responded, non-outlier trials and percentage of correct response for every experimental condition are reported in Table 1.

**TABLE 1:** **Mean reaction times and accuracy for the two groups as a function of SOA, facial expression, and cue-target spatial congruency**.

		200 ms SOA					
		Disgust		Fear		Anger	
		Congruent	Incongruent	Congruent	Incongruent	Congruent	Incongruent
Snake	RT	566 (81) ms	588 (79) ms	574 (84) ms	597 (74) ms	574 (63) ms	591 (85) ms
Phobics	% Correct	95.58 (5.8)	96.05 (4.94)	95.05 (6.35)	94.58 (5.06)	96.79 (5.06)	95.53 (6.14)
Control	RT	553 (67) ms	572 (63) ms	554 (63) ms	568 (69) ms	550 (62) ms	560 (53) ms
Group	% Correct	96.6 (3.08)	95.5 (4.07)	97.85 (3.2)	96.2 (4.57)	97.2 (3.69)	96.4 (4.08)
				**500 ms SOA**			
Snake	RT	546 (67) ms	577 (77) ms	555 (82) ms	597 (104) ms	551 (76) ms	584 (80) ms
Phobics	% Correct	96.16 (4.67)	95.47 (5.56)	94.32 (7.77)	93.42 (6.27)	97.68 (4.07)	94.84 (5.62)
Control	RT	511 (51) ms	534 (62) ms	513 (51) ms	544 (66) ms	509 (45) ms	534 (67) ms
Group	% Correct	93.5 (8.79)	93.65 (5.86)	95.8 (3.78)	92.55 (4.38)	95.6 (3.87)	93 (7.19)

Standard deviations are in parentheses.

Mean correct RTs were entered into a 2 × 2 × 3 × 2 ANCOVA with Congruency (congruent vs. incongruent), SOA (200 vs. 500 ms) and Expression (fear vs. disgust vs. anger) as *within participant* factors, and Group (SP vs. NP) as *between participant* factor. Trait anxiety scores were entered as covariate.

There was a significant Congruency main effect, *F*(1,36) = 12.81, *p* = 0.001, η^2^ = 0.26, 90% CI [0.08, 0.43], with faster responses on congruent than on incongruent trials. A significant SOA × Group interaction, *F*(1,36) = 13.25, *p* = 0.001, η^2^ = 0.27, 90% CI [0.08, 0.43], showed that, although the long SOA produced globally faster RT, this difference was less pronounced for SP (11 ms) as compared to NP group (37 ms). The Congruency × Expression interaction was also significant, *F*(2,72) = 3.21, *p* = 0.046, η^2^ = 0.08, 90% CI [0.001, 0.18]. The gaze-cuing effect was significant for all three expressions (*p*s < 0.001), but seemed more pronounced for fearful faces (28 ms) and less pronounced for angry faces (21 ms), with disgusted faces in the middle (24 ms). A significant Congruency × Group interaction also emerged, *F*(1,36) = 4.3, *p* = 0.045, η^2^ = 0.11, 90% CI [0.001, 0.27], showing that the cuing effect was significant for both groups (*p*s < 0.001), but more pronounced for SP (31 ms) as compared to NP group (18 ms). No significant interactions of trait anxiety with the critical experimental factors were observed. Because the present pattern emerged while taking the role of anxiety into account, this in turn ensures that trait anxiety did not play any relevant role in the present data.

With regards to accuracy, an ANCOVA with the same factors as above was conducted on the percentage of correct responses calculated on all trials. The only significant effect involved SOA, *F*(1,36) = 6.8, *p* = 0.01, η^2^ = 0.16, 90% CI [0.02, 0.33] (see Table 1). The pattern of accuracy data makes the possibility of a speed-accuracy tradeoff unlikely.

## Discussion

The aim of this study was to investigate the role of facial expressions in gaze-driven orienting of attention in animal phobia. Although the presence of an attentional bias for stimuli representing the phobic object is well documented in animal phobia (Öhman, 2008), only a few studies have investigated the existence of a processing bias in this disorder using paradigms in which the feared animal was not eventually presented. Among these, recent studies have reported a processing bias for emotional facial expressions (Wright et al., 2003; Sarlo and Munafò, 2010), that was limited to fear and disgust in early processing stages, and extended to generally negative expressions during later stages (Sarlo and Munafò, 2010). Emotional faces are highly salient stimuli that can signal the presence of relevant objects in the environment. The bias for emotional faces detected in animal phobics may depend on the fact that these stimuli could signal the presence of the feared animal in the surroundings, triggering a hypervigilant processing mode aimed at quickly localizing the phobic stimulus in the environment. This could be especially true for fearful and disgusted expressions, that phobic individuals relate to the phobic object (de Jong et al., 2002; Olatunji et al., 2007).

Starting from this hypothesis, we conducted an experiment using a gaze-cuing paradigm to test whether SP participants would show an attentional bias that is sensitive to emotional expressions. The literature suggests that emotional facial expressions can modulate this phenomenon, especially in highly anxious and highly fearful people (Mathews et al., 2003; Tipples, 2006; Fox et al., 2007). We used a paradigm characterized by high ecological validity, in which negative facial expressions changed dynamically after gaze movement, a situation that mimics contexts typically faced in everyday life (Graham et al., 2010). This is a very important feature, because the vast majority of previously published studies used procedures in which the emotional facial expression was statically presented on screen right from the beginning (e.g., Hietanen and Leppänen, 2003), or in which the gaze cue appeared after the emotional expression (e.g., Mathews et al., 2003; Fox et al., 2007) or simultaneously with it (e.g., Tipples, 2006).

The present results, consistent with previous studies (see Frischen et al., 2007), showed that all participants responded more quickly to targets appearing in the location signaled by the eyes of a centrally presented face than to targets appearing in the opposite direction. These results confirm the notion that the gaze of others can reflexively push attention in an observer. Importantly, gaze direction was overall more effective in driving attention for SP as compared to NP individuals. Moreover, gaze cuing was not selectively enhanced for disgusted and fearful faces in SP individuals as hypothesized, but rather non-specifically enhanced in SP as compared to NP participants, irrespective of both facial expression and SOA. Such effects cannot be ascribed to differences in trait anxiety, because the role of anxiety was statistically controlled for in the analysis. As for state anxiety, we did not control this variable because, to the best of our knowledge, there is no indication from the literature that phobic individuals should be more state-anxious than NP individuals, unless they are going to be exposed to the phobic stimulus. Our participants were aware that they would not be exposed to phobic stimuli, and thus we had no reason to assume that the two groups would differ on state anxiety.

Overall, the present results suggest that the attentional bias for emotional expressions in SP individuals appears relatively early. This is consistent with the findings observed by Sarlo and Munafò (2010), who reported a general hypervigilance for negative emotional expressions in SP individuals in a time window from 200 to 400 ms after the emotional face appeared on screen. The fact that SOA did not interact with gaze cuing is still consistent with the view that hypervigilance to emotional faces is not a long-lasting phenomenon because in the implemented experimental procedure emotional expression appeared 100 ms after the gaze cue.

According to the literature, attentional bias is hypothesized to consist of three different mechanisms: an enhanced orienting to threat, followed by a delayed disengagement from it, and possibly by cognitive avoidance in a subsequent stage. Whether all three of these mechanisms are present is still under debate and is still an active research topic (see Cisler and Koster, 2010, for a review). Our result may be consistent with both enhanced orienting and delayed disengagement, as these mechanisms cannot be discerned in gaze-cuing tasks. However, we did not find evidence for avoidance, which would have caused slower RTs or lower accuracy to gazed-at targets at the longer SOA. This lack of avoidance could be due to the fact that participants knew that no phobic stimuli were to be displayed in this paradigm. To investigate avoidance with a gaze-cuing paradigm, future studies may address this issue directly, by including blocks with phobic targets and blocks with neutral targets (cf. Friesen et al., 2011).

According to a recent review by Heeren et al. (2013), the current theoretical accounts on attentional bias in anxiety can be divided in two broad categories: valence specific models (e.g., Beck and Clark, 1997; Mathews and Mackintosh, 1998; Mogg and Bradley, 1998) and attentional control models (e.g., Bishop et al., 2004; Eysenck et al., 2007; Bishop, 2008). Valence specific models posit that attentional bias is due to a distorted initial threat appraisal, such that people with anxiety disorders classify ambiguous or mildly threatening stimuli as highly threatening. This would cause enhanced orienting and/or delayed disengagement to these stimuli. Attentional control models, on the other hand, ascribe attentional bias to a deficit in top-down attentional control, such that individuals with anxiety disorders are impaired in inhibiting stimulus-driven attentional shifts away from their goals. Our results could be interpreted as consistent with both accounts: on the one hand, following the valence specific models, the hypervigilance observed in our study could indicate that facial expressions were interpreted by SP participants as threat cues, and this produced a heightened attention to their gaze directions in search for threat. On the other hand, our results could also indicate a greater difficulty in ignoring the eye-gaze, that would be compatible with attentional control accounts. The fact that SP participants showed a more pronounced gaze cuing effect, not only to disgusted and fearful faces that could be cues to a phobic threat, but to angry faces as well, seems to provide stronger support for this second explanation, as it is difficult to assume that angry faces could indicate the presence of a snake in the environment. However, another possible explanation is that in our study two of the facial expressions employed were phobia-related, and this might have caused in SP participants a generalized hypervigilance that may have increased attention to gaze direction for every emotional expression.

In any case, our current results indicate that attentional bias in animal phobia is not as strictly specific as commonly thought, and thus it would be interesting to investigate the extent of its specificity by testing whether it can only be observed in contexts where stimuli that are somehow related to the phobic object are present (as phobia-related facial expressions) or also for other types of emotional stimuli in non-phobic situations. This would also provide new information concerning the specificity of attentional biases in anxiety disorder, a topic which is of special interest in current attentional bias research (e.g., Pergamin-Hight et al., 2015).

Before proceeding to the final conclusions, it is important to acknowledge that this study has several limitations. First of all, the sample size was relatively small, due to the strict selection criteria, and this forces us to consider the results with caution as the analyses we used are complex and may be underpowered with the present samples. Moreover, the effect sizes of our main results are small. Further research may be required to re-test these effects and generalize them to other experimental contexts. Secondly, the sample was exclusively composed of young women and thus the result may not generalize to men or to other age groups. However, this is the first study conducted with specific phobics using a gaze cuing paradigm. Hence, since the topic of attentional biases in animal phobics in the absence of phobic stimuli is little explored, our results can be of potential interest regarding the understanding of attentional biases, their specificity, and their role in anxiety disorders.

The fact that SP participants showed a different sensitivity to eye gaze in an emotional context as compared to controls is not consistent with the classical idea of animal phobia, according to which these individuals suffer from processing biases only when the feared stimulus is present or expected to appear (Öhman, 2008). In contrast, the present study seems to confirm the existence of an attentional bias in SP individuals in the absence of phobic stimuli in line with previous studies (Wright et al., 2003; Sarlo and Munafò, 2010) and also seems to indicate that animal phobics are subject to a monitoring of the environment that is not restricted to conditions in which phobic stimuli are actively expected to appear, similarly to what happens to people who suffer from clinical anxiety, who continuously monitor the environment even in safe situations (Mogg and Bradley, 1998, 2005). On the contrary, the present results show that animal phobics do not need to be in a situation where the feared animal is likely to appear to suffer from heightened vigilance and to start monitoring the environment: this state can also be triggered by NP, socially-salient emotional stimuli in conditions that should be safe for the phobic individual.

The present findings, if tested and extended to other animal phobias and other populations (varying the gender and the age-range), may also provide useful insights for the treatment of animal phobia, by providing information for the creation of appropriate attentional bias modification trainings (ABMT) such as those that are already being proposed for the treatment of anxiety (Hakamata et al., 2010), social phobia (Amir et al., 2008; Heeren et al., 2012b), and spider phobia (Reese et al., 2010).

In conclusion, our study provides new insights on specific phobias that could be useful for its treatment and indicates that research on specific phobias should not be limited to symptom provocation paradigms, because this would preclude the unveiling of some of the attentional mechanisms that underlie this disorder.

## Author Contributions

All authors provided substantial contributions to the conception or design of the work; or the acquisition, analysis, or interpretation of data for the work; and contributed in drafting the work or revising it critically for important intellectual content; and approved the final version for publication; and agreed to be accountable for all aspects of the work in ensuring that questions related to the accuracy or integrity of any part of the work are appropriately investigated and resolved. This research was conducted with the assistance of Ms. Martina Papa and Ms. Alessandra Tafuro, who helped in administering the task to the participants. The authors are grateful to S. Gareth Edwards for his valuable comments on a previous draft.

### Conflict of Interest Statement

The authors declare that the research was conducted in the absence of any commercial or financial relationships that could be construed as a potential conflict of interest.

## Abbreviations

NP: non-phobic
SP: snake phobic.
